# AAV-based dual-reporter circuit for monitoring cell signaling in living human cells

**DOI:** 10.1186/s13036-017-0060-9

**Published:** 2017-06-05

**Authors:** Zhiwen Zhang, Zachary Stickney, Natalie Duong, Kevin Curley, Biao Lu

**Affiliations:** Department of Bioengineering, Santa Clara University, 500 El Camino Real, Santa Clara, CA 95053 USA

**Keywords:** AAV, NF-κB, AP-1, TNFα, GFP, Firefly luciferase

## Abstract

**Background:**

High-throughput methods based on molecular reporters have greatly advanced our knowledge of cell signaling in mammalian cells. However, their ability to monitor various types of cells is markedly limited by the inefficiency of reporter gene delivery. Recombinant adeno-associated virus (AAV) vectors are efficient tools widely used for delivering and expressing transgenes in diverse animal cells in vitro and in vivo. Here we present the design, construction and validation of a novel AAV-based dual-reporter circuit that can be used to monitor and quantify cell signaling in living human cells.

**Results:**

We first design and construct the AAV-based reporter system. We then validate the versatility and specificity of this system in monitoring and quantifying two important cell signaling pathways, inflammation (NFκB) and cell growth and differentiation (AP-1), in cultured HEK293 and MCF-7 cells. Our results demonstrate that the AAV reporter system is both specific and versatile, and it can be used in two common experimental protocols including transfection with plasmid DNA and transduction with packaged viruses. Importantly, this system is efficient, with a high signal-to-background noise ratio, and can be easily adapted to monitor other common signaling pathways.

**Conclusions:**

The AAV-based system extends the dual-reporter technology to more cell types, allowing for cost-effective and high throughput applications.

**Electronic supplementary material:**

The online version of this article (doi:10.1186/s13036-017-0060-9) contains supplementary material, which is available to authorized users.

## Background

Cell signaling plays a critical role in regulating cellular functions that are essential for normal development, stem cell renewal, and tissue regeneration [1–4]. Conversely, abnormal signaling may cause birth defects and contribute to the pathogenesis of many human disorders including cancer, cardiovascular and neurological diseases, autoimmunity, infection and inflammation [5–10]. In humans, a plethora of signaling molecules, cellular receptors and regulatory pathways are uniquely employed to provide cell-specific responses to various environmental stimuli such as changing local concentrations of growth factors, or defense and inflammatory cytokines [11–13]. Since cells may be regulated or perturbed due to altered metabolic status or receptor levels, methods to accurately report cell signaling activities at individual cell levels would be useful [14, 15]. To meet such research needs, various biosensors have been invented, and those biosensor-based reporters have improved sensitivities and specificities as compared to those of biological- or biochemical-based assays [16–18]. Among the available biosensors, the biocompatible green fluorescent protein (GFP) and firefly luciferase (Luc) are frequently chosen as the molecular reporter, because of their sensitivity and high signal-to-noise ratio [19–23]. Each type of reporter may reveal certain unique molecular events. For example, while GFP provides a better means for analyzing individual cell response [24, 25], luciferase may be a handy tool for signal quantification [26, 27]. Together, the dual reporter of GFP and luciferase enable delineation of a full and precise picture of cell signaling in living systems [28–31].

Previously, we have built and validated a dual-reporter system that significantly improves the system capacity and performance in studying cell signaling with live cell visualization and high through-put quantification [28, 29, 32, 33]. However, the established system was initially built into a conventional plasmid vector, which only can be used in certain cell types by transfection protocols. In order to expand to more cell types and potentially into living tissues or animals, in this study we explore the possibility of using a viral vector to accommodate the dual-reporters. To achieve this goal, we chose the recombinant adeno-associated virus (AAV) vector, which has a number of advantages over other viral vectors such as retro-virus and adeno-virus. First, the small AAV vectors are most efficient in delivering transgenes in diverse cell types both in vitro and in vivo [34–36]. They can transduce both dividing and non-dividing cells, and they have a prolonged period of expression [35, 37]. Additionally, numerous serotypes provide a handy means to achieve tissue-specific delivery to desired cell types [34, 37, 38]. Second, AAV vectors contain small non-coding viral sequences, namely the inverted terminal repeat (ITR), making them less likely to interfere with the reporter system [35]. In contrast, both retro- and adeno-viral vectors have more virus coding/non-coding sequences, which may lead to high levels of background noise or the possibility of interfering with functions of the reporter system [39, 40]. Third, AAV are nonpathogenic and have proven to be safe by numerous clinical trials [41]. Finally, AAV vectors are technically flexible in applications. Because AAV vector by nature is not very different from regular plasmid vector, we can use simple transfection protocols to carry out experiments in easy-to-transfect cells.

Here, we present the development of a novel AAV-based dual reporter system for cell signaling studies. We design and construct the AAV-based dual-reporter and examine the sensitivity and specificity of the system using cultured HEK293 cells. We subsequently demonstrate the success of this system in monitoring of inflammatory and cell growth signaling pathways in two human cell lines. Together, this novel AAV-based signaling reporter system extends the dual-reporter technology to more cell types, allowing for cost-effective and high throughput applications.

## Methods

### Materials and reagents

Human recombinant TNFα was purchased from R&D Systems (Minneapolis, MN). Phorbol-12-myristate 13-acetate (PMA) was purchased from Sigma (St. Lois, MO). The cell culture lysis reagent and luciferase assay substrate were purchased from Promega (Madison, WI). Fetal bovine serum (FBS) was purchased from ThermoFisher Scientific (Waltham, MA). Recombinant AAV-DJ vector and helper free packaging system were purchased from Cell Biolabs (San Diego, CA).

### Reporter construction

The dual reporter cassette was built into an AAV helper virus-free system using a fusion strategy as previously reported [33, 42]. The reporter cassette was flanked by the inverted terminal repeats (ITRs) derived from AAV. The dual-reporter cassette was configured from the 5’end to the 3’end as follows: the multiple cloning sites (MCS) to accommodate transcription factor response elements (TREs), a minimal CMV promoter (mCMV), GFP-2A-Firefly-luciferase, then a poly adenylation signal (Poly A). To assess the background noise and detection range, two additional reporters were also similarly built. These include a promoterless reporter and a reporter containing a full-length CMV promoter and enhancer. For cell signaling reporters, 4–8 repeats of the TREs were cloned into the MCS at the 5’ end adjacent to mCMV. All final constructs were subjected to double-stranded DNA sequencing and their sequences from ITR to ITR were provided in Additional file 1.

### Cell culture, transfection, and transduction

Human Embryonic Kidney 293 (HEK293) and human breast cancer cell MCF-7 were purchased from Alstem (Richmond, CA) and ATCC (Manassas, VA) respectively. All cells were maintained in high glucose Dulbecco’s modified Eagle’s medium (DMEM) supplemented with 10% fetal bovine serum (ThermoFisher Scientific), 2 mMGlutaMAX (Life Technologies) and 1% penicillin-streptomycin 100 U/ml (Life Technologies) and cultured at 37 °C with 95% air and 5% CO_2_. At ~80% confluence, cells were dissociated with 1X trypsin-EDTA and passed at ratio of 1:4.

Both transfections and transductions were performed in 6-well plates unless otherwise stated. Briefly, cells at ~60–70% confluency were transfected by plasmid DNA (1 ~ 2.5 μg/well) mixed with either Lipofectamine (Thermo Fisher Scientific) or FuGENE 6 transfection reagent (Promega) for indicated periods of time. Alternatively, cells were transduced by recombinant AAV with 0.5 ~ 1 of multiple of infection (MOI) for 24 h.

### Production of recombinant AAV from HEK293 cells

Recombinant AAV production was based on transfection of HEK293 cells by three plasmids using Lipofectamine-mediated transfection. Typically, cells growing on 15-cm culture dishes were transfected with a DNA mix composed of AAV reporter, Rep and Cap plasmid, and AAV helper plasmids (Cell Biolabs, Inc. San Diego, CA). Twenty-four hours after transfection and incubation, culture media were switched to complete growth media for an additional 48 h. The AAV particles were prepared from the culture supernatant using an AAV concentration reagent according to the manufacture’s manual (System Biosciences, Mountain View, CA). The AAV reporter vectors were packaged using AAV-DJ capsids, which were derived from 8 different wild-type viruses [36]. We chose AAV-DJ viral serotype as they have a broad tropism and efficient transduction in vitro and in vivo [36].

### AAV titration and multiplicity of infection (MOI) determination

Both green cell fluorescent assays and PCR were used to determine the MOI as reported [38, 43]. For the green cell fluorescent assays, HEK293/MCF-7 cells grown on 12-well plates were infected with serial dilutions of CMV-GFP-2A-Luciferase positive control virus. Seventy-two hours later, cells infected with GFP-positive virus were visually scored using a fluorescence microscope, and the viral MOI was determined by GFP positive cells. The AAV-reporter’s MOI was estimated by the relative copy number of recombinant virus verses that of the positive control viruses.

### Firefly luciferase activity assay

Luciferase activity was measured by a Microplate luminometer (Applied Biosystems) as reported previously [28, 33]. Briefly, cells were lysed with a Passive Lysis Buffer (Promega) and cell lysates were cleared by centrifugation at the 12,000 rpm for 2 min. The supernatants were saved for Luc activity assay. For Luc quantification, 100 μL of substrate was added to 10 μL of sample supernatant, according to the user’s manual (Promega, USA).

### Data collection & presentation

For live cell monitoring, cultured cells were monitored under a fluorescent microscope. Images were taken at indicated time-points using the same exposure condition within the group of comparison. For luciferase reporter assay, data are presented as the mean ± SD (n = 3), unless stated otherwise.

## Results and Discusssion

### Design and construction of AAV-based dual-reporters for pathway monitoring

**Fig. 1:**
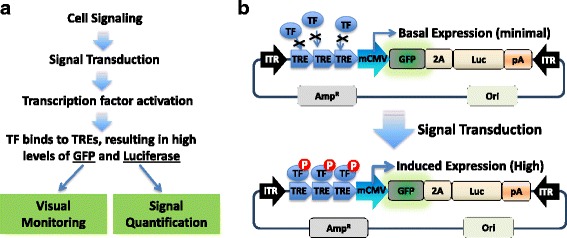
System design of AAV-based dual reporters for monitoring cell signaling. **a** Visualization and quantification of cell signaling with GFP and firefly luciferase (Luc). Cell signaling is illustrated as sequential events, including the increase in amount of signaling molecule, binding of signaling molecule to its receptor, signaling transduction, activation of transcription factor (TF), and final activations of reporter genes (GFP and Luc). **b** Schematic illustration of dual-reporter design and construction. Specific signaling reporters can be constructed by inserting the transcription factor responding elements (TRE) immediately upstream of the minimal CMV promoter (mCMV). These cell-signaling specific reporters can trace transcription activities in mammalian cells depending on the status of TFs with minimal expression of reporters in the absence of TFs (*upper panel*) and a high expression of the reporters upon the activation of TFs (*lower panel*)

The ability to monitor and quantify the temporal activation of a pathway signaling in living cells, both at the individual cell level and within a tissue, is highly desirable. To achieve this goal, we generated a genetic circuit that consists of a transcription control unit (TRE and mCMV) and a dual-reporter fusion (GFP and Luc) (Fig. 1a, upper panel). Under this configuration, the transcription control unit of TRE will respond to the binding of their corresponding activated transcription factors (TFs), resulting in reporter gene expression (Fig. 1a, lower panel). Because different cell signaling pathways may activate distinct sets of TFs, carefully chosen TRE sequences will allow construction of different circuits for specific signaling detection. For example, to detect and monitor inflammation, one may insert NFκB response elements as TREs (Fig. 1b). This NFκB genetic circuit may respond to inflammatory signaling molecules in its environment. Ideally, this circuit responds only to inflammatory signaling molecules, such as proinflammatory cytokine tumor necrosis factor alpha (TNFα) [44–46].

Accordingly, we have built the following cell signaling circuits, which can potentially monitor activations of signaling pathways, including inflammation (NFκB) and cell growth and differentiation (AP1). Normally a 4 ~ 6 tandem repeats of TREs are joined together via a 6-bp spacer. These reporting circuits were constructed into an AAV-based vector system by providing two short flanking sequences of inverted terminal repeats (ITR) to allow AAV packaging. The detail sequences from ITR to ITR of the described reporters are provided in the Supplementary sequences (Additional file 1).

### Characterization of background noise, detection range and signal-to-noise ratio

**Fig. 2:**
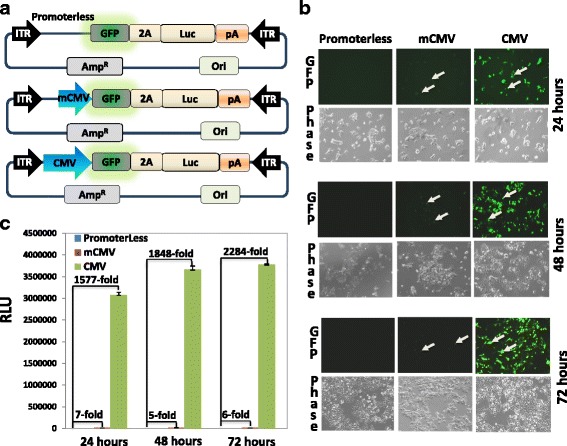
Construction of AAV-based dual reporters and their functional characterization. **a** Schematic illustration of 3 AAV-based dual reporters used for system validation. All reporters have the basic configuration, including the AAV-related ITR sequences for AAV-production and packaging, the two reporter genes GFP and Luc separated by 2A sequences (self-cleavage peptide). The dual-reporters are either promoterless or under the control of mCMV or fCMV, as shown from top to bottom. **b**The GFP expression (*green*) in HEK293 cells were recorded with a fluorescence microscope following transfections of either a promoterless, a mCMV, or a fCMV-driven reporter at 24 h (*top panel*), 48 h (*middle panel*) and 72 (*lower panel*). *Arrows* indicate GFP-positive cells. **c** The Luc expression in HEK293 cells were determined by luciferase assay following transfections of three reporters at same time points. The luciferase activities are expressed as relative light units (RLU), normalized against protein input, and presented as fold increase over untreated control (mean ± SD, *n* = 3)

To evaluate the background noise, detection range and signal-to-noise ratio of this AAV-based dual-reporter platform, we constructed three additional reporters: 1) a promoterless (background), 2) a minimal cytomegalovirus promoter (mCMV), and 3) a full-length CMV promoter (fCMV) (Fig. 2a). We delivered these reporters into HEK293 cells by transfection and observed the expression of both GFP (Fig. 2b) and firefly-luciferase activities (Fig. 2c) up to 72 h. As expected, most cells from the promoterless reporter group remained GFP negative for up to 72 h, indicating very little background noise of this AAV-based dual-reporter circuit (Fig. 2b, left panel). For minimal promoter group, a few GFP-positive cells were detected, consistent with the minimal transcriptional activities of mCMV (Fig. 2b, middle panel). In contrast, when transfected with the fCMV plasmid, ~80% of cells showed strong GFP-positivity in a time-dependent manner (24 ~ 72 h) (Fig. 2b, right panel). These data demonstrated that GFP can serve as visual clue of signaling activity, correlating well to the activation of the monitored transcriptional activity.

We next examined Luc activities of transfected cells in parallel, to see whether the Luc activities were in line with GFP-positivity and expression levels. Consistent with our image findings, while the promoterless group showed a very low background level (24 ~ 72 h), the minimal promoter group showed a small but consistent ~6-fold increase in Luc activity (Fig. 2c). In contrast, a robust ~1577-2284-fold increase in Luc activity was detected for the fCMV promoter (24 ~ 72 h) (Fig. 2c). These results confirm that the AAV-based dual reporter system has superior sensitivity with low levels of background noise and a very high signal-to-noise ratio of ~2000:1.

### The AAV-based dual-reporter system provides a specific platform for monitoring cell signaling

**Fig. 3:**
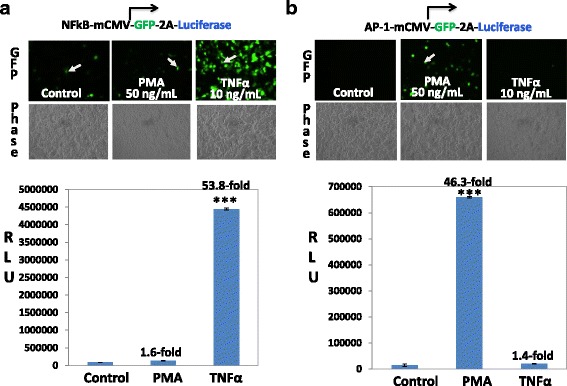
The specificity of the AAV-based dual-reporters using transfection protocol. **a** HEK293 cells were transfected with NF-κB reporters for 24 h. Cells were then switched to low serum medium in the presence or absence of either TNFα (10 ng/mL) or PMA (50 ng/mL) for 24 h. The expression of GFP (images, *upper two panels*) and Luc activities (the l*ower graph*) are shown. **b** In a separate set of experiments, cells were transduced with AP-1 reporters for 24 h, followed by the treatment of either TNFα (10 ng/mL) or PMA (50 ng/mL) for additional 24 h. The expression of GFP (images, *upper two panels*) was recorded. *Arrows* indicates the GFP-positive cells. The luciferase activities are expressed as relative light units (RLU), normalized against protein input, and presented as fold increase over untreated control (mean ± SD, *n* = 3). *** *P* < 0.001, Student *t*-Test versus controls

We next examined whether the AAV-based dual-reporter strategy could serve as a specific platform for monitoring various signaling pathways. We conducted a side-by-side study on two well-characterized pathways, inflammation (NFκB) and cell growth& differentiation (AP-1). These pathways play important roles in cellular function, and they can be activated and regulated to well-defined signaling molecules such as proinflammatory TNFα and cancer promoting reagent PMA, respectively. Accordingly, we designed and constructed two reporters using inflammation (NFκB) and cellular growth (AP-1) responding elements. We transfected cultured HEK293 cells with these reporters to determine the effects of TNFα or PMA on reporter gene activation. As shown in Fig. 3a, treatment with 10 ng/mL TNFα resulted in a robust increase in intensity of GFP in inflammatory NFκB reporter group. In parallel, a 53.8-fold increase in luciferase activity was observed, supporting a true activation of the dual-reporter (Fig. 3a). In contrast, only a few GFP-positive cells were seen in either control reporter cells or cells treated with cancer promoting reagent PMA (Fig. 3a, top panel). These results suggest that the inflammatory reporter has a specific response in detecting inflammation-related signaling. Similarly, following transfection of cells with AP-1 reporter plasmid, PMA treatment (50 ng/mL) resulted in a marked increase in GFP intensity, paralleled with a 46.3-fold increase in Luc activities (Fig. 3b). Because neither mock-transfection nor TNFα exerted a significant effect on expressions of GFP or Luc under the same experimental condition (Fig. 3b), we conclude that the AAV-based dual-reporter system provides a specific platform for monitoring cell growth and differentiation signaling in human cells. Together, our results validate the strategy of using AAV-based dual-reporter system for monitoring various cell signaling pathways.

### Monitoring cell signaling by transduction protocol

**Fig. 4:**
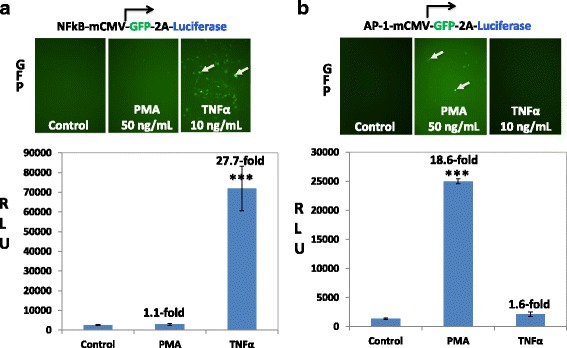
The specificity of AAV-based reporter using transduction protocol. **a** HEK293 cells were transduced with either NFκB (**a**) or AP-1 (**b**) reporter AAV (MOI = 1) for 24 h. Cells were then switched to low serum medium in the presence or absence of TNFα (10 ng/mL) or PMA (50 ng/mL) for 24 h. The expression of GFP (images, *upper two panels*) and Luc activities (the *lower graph*) were examined by fluorescence microscope or luciferase assay respectively. The luciferase activities are expressed as relative light units (RLU), normalized against protein input, and presented as fold increase over untreated control (mean ± SD, *n* = 3). *** *P* < 0.001, Student *t*-Test versus controls

After successful monitoring of cell signaling pathways using a transient transfection protocol, we next examined whether the AAV-based reporters could be packaged into recombinant viral particles for monitoring pathway signaling in transduced cells. We used the following three plasmids to generate all recombinant reporter viruses: AAV-based reporter, helper plasmid and rep-cap plasmid. Typically, one MOI equivalent viruses was used to transduce culture HEK293 cells for 24 h. Following the transduction, cells were switched to medium containing low level of serum with either TNFα or PMA for pathway activation. As expected, the inflammatory NF-κB dual-reporter exhibited a robust increase in GFP-intensity and Luc activities (27.7 fold over control) in responding to the treatment of TNFα but not PMA (Fig. 4a). Similarly, the AP-1 dual reporter exhibited an increase in GFP intensity (Fig. 4b, top panel) and Luc activities (18.6-fold over the non-treatment control) following the treatment of PMA but not TNFα (Fig. 4b). In both cases, the control group exhibited a limited GFP intensity and Luc activities (Fig. 4). These results validate our AAV-based dual-reporter in cell signaling pathway monitoring in transduced cells. Together, our data strongly support a notion that this new system can serve as versatile platform for pathway signaling monitoring, using either simple transfection or viral transduction protocol.

### TNFα activates inflammatory pathway in a dose- and time-dependent manner

**Fig. 5:**
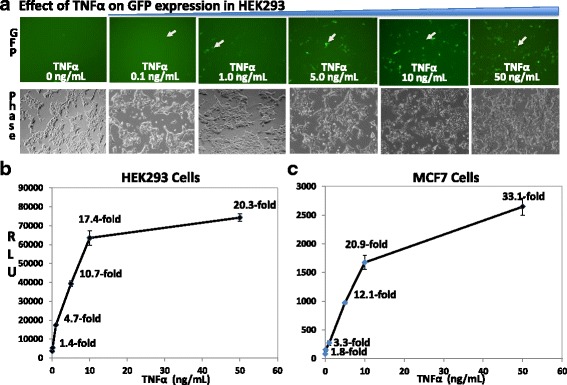
Dose-response of NF-κB reporter in HEK293 and MCF-7 cells. **a** Following transduction with NF-κB reporter virus (MOI = 1) for 24 h, HEK293 cells (**a**) or MCF-7 cells (**c**) were treated with increasing concentration of TNFα (0, 0.1, 1.0, 5.0, 10, and 50 ng/mL) for 24 h, and GFP expression was recorded. **b** The luciferase activities were determined by luciferase assay using cell lysates, and Luc activities expressed as relative light units (RLU), normalized against protein input, and presented as fold increase over untreated control (mean ± SD, *n* = 3)

To further characterize the monitoring function of the dual-reporter systemically in transduced cells, we carried out dose-response and time-course studies on the AAV-based inflammatory reporter and examined its ability to monitor the effects of TNFα on two human cell models. We first conducted a dose-response experiment on HEK293 cells. As shown in Fig. 5a, as low as 0.1 ng/mL, TNFα can increase the GFP signal, and the percentage of GFP-positive cells increases with the dosage of TNFα up to 10 ng/mL. A higher dosage of 50 ng/mL exhibited an attenuated trend. Consistent with GFP results, we observed a similar dose-dependent increase in Luc activity (Fig. 5b), with a marked 20.3-fold increase in luciferase activity in cells treated with 50 ng/ml TNFα as compared to the untreated controls.

To further confirm the sensitivity and dose range of the dual reporter, we performed a dose-response experiment on the human breast cancer MCF-7 cells. Similarly, we observed a steady increase in the Luc activities in response to increasing concentrations of TNFα (Fig. 5c). The response in MCF-7 appeared more robust than those in HEK293 cells, as higher fold-increase in Luc values at each dose-point. However, the tread of response of both cell types remained the same. Together, our results strongly indicate that the dual-reporter can serve as a sensitive tool to visualize and quantify pathway signaling in various types of mammalian cells. Importantly, the dose–response data are consistent with previous reporters and TFNα dose-ranges relevant to inflammation and sepsis [44–46].

**Fig. 6:**
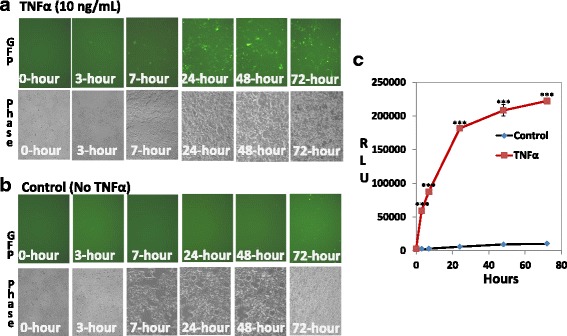
A time-course study of NF-kB reporter in transduced HEK293 cells. Following transduction with NF-κB reporter virus (MOI = 1) for 24 h, HEK293 cells were switched to low serum medium in the presence or absence of TNFα (10 ng/mL) for 0, 3, 7, 24, 48, and 72 h. **a** GFP expression for the TNFα treatment group. **b**. GFP expression for the untreated control group. **c** At the same time points, luciferase assays were conducted and luciferase activities were plotted as relative light units (RLU), normalized against protein input (mean ± SD, *n* = 3). *** *P* < 0.001, Student *t*-Test versus controls

One important advantage of the dual-reporter system is that GFP enables real-time monitoring of pathway activation in monitored cells, while luciferase allows signal quantification. To further test the dynamic response of the NFκB dual-reporter, we carried out a time-course study on the effects of TNFα on HEK293 cells. We chose a dose of 10 ng/mL TNFα since this dose produced a robust inflammation response in our transduced HEK293 cells (as described above). As shown in Fig. 6a, as early as 3 h after TNFα treatment, GFP-positive cells began to appear; with time GFP-intensity steadily increased up to 72 h. In contrast, no GFP-positive cells were observed in the absence of TNFα (Fig. 6b). In agreement with GFP expression, we found a corresponding time-dependent increase in luciferase activity in the TNFα treatment group but not in the non-treatment control group (Fig. 6c). These results show a strong correlation between the visual assessment by GFP and Luc quantification. Our data also confirm the specificity of the dual reporter in dynamically monitoring cell signaling in living cells.

Using AAV-based inflammatory reporters, we were able to dynamically and quantitatively monitor the NFκB pathway activation by TNFα in living HEK293 and MCF-7 cells. The reporter demonstrated high specificity and excellent signal-to-noise ratio. The fact that they can be used in common applications such as time-course and dose-response studies supports the usefulness of this novel experimental tool.

## Conclusions

We have designed and constructed a novel AAV-based dual-reporter system for monitoring cell signaling activities that are critical for biology and disease processes. This system combines two distinctive biological reporters of GFP and Luc, enabling both visualization and quantification of signaling activities. Because the AAV-based vectors are simple, safe, and effective in transducing diverse animal cells in vitro and in vivo, the new AAV-based system will extend the dual-reporter technology to more cell types, allowing for cost-effective and high throughput applications.

## Additional file

Additional file 1: Supplementary sequences. (DOCX 19 kb)

## Abbreviations

AAV: Adeno-associated virus
AP1: Transcription factor AP-1
CMV: Full-length cytomegalovirus promoter
GFP: Green fluorescent protein
HEK293: Human embryonic kidney 293 cells
HTP: High-throughput
ITR: Inverted terminal repeats
Luc: Luciferase
mCMV: minimal CMV promoter
NF-κB: Nuclear factor kappa-light-chain-enhancer of activated B cells
PMA: Phorbol-myristate-acetate
TF: Transcription factor
TNFα: Tumor necrosis factor alpha
TRE: Transcription factor response elements
