# Supplementary material for: AAV-based dual-reporter circuit for monitoring cell signaling in living human cells
Source: J Biol Eng. 2017 Jun 5;11:18. doi: 10.1186/s13036-017-0060-9 (PMC5458475; doi:10.1186/s13036-017-0060-9)
Supplement: Additional file 1: — Supplementary sequences. (DOCX 19 kb) [file 13036_2017_60_MOESM1_ESM.docx]

Additional file 1

>ITR-Promoterless-GFP-T2A-Luc-PolyA-ITR to monitor inflammation pathway signaling (NF-kB)

ttggccactccctctctgcgcgctcgctcgctcactgaggccgggcgaccaaaggtcgcccgacgcccgggctttgcccgggcggcctcagtgagcgagcgagcgcgcagagagggagtggccaactccatcactaggggttccttgtagttaatgattaacccgccatgctacttatctacgtaacagatctgatatcacgcgtgtacactagtaccggtcctaggctcgagctccaagtgcaggggaaagaatagtagacataatagcaacagacatacaaactaaagaattacaaaaacaaattacaaaattcaaaattttatcgatgaattcctctagaaactagttgggatccgccaccatgcccgccatgaagatcgagtgccgcatcaccggcaccctgaacggcgtggagttcgagctggtgggcggcggagagggcacccccgagcagggccgcatgaccaacaagatgaagagcaccaaaggcgccctgaccttcagcccctacctgctgagccacgtgatgggctacggcttctaccacttcggcacctaccccagcggctacgagaaccccttcctgcacgccatcaacaacggcggctacaccaacacccgcatcgagaagtacgaggacggcggcgtgctgcacgtgagcttcagctaccgctacgaggccggccgcgtgatcggcgacttcaaggtggtgggcaccggcttccccgaggacagcgtgatcttcaccgacaagatcatccgcagcaacgccaccgtggagcacctgcaccccatgggcgataacgtgctggtgggcagcttcgcccgcaccttcagcctgcgcgacggcggctactacagcttcgtggtggacagccacatgcacttcaagagcgccatccaccccagcatcctgcagaacgggggccccatgttcgccttccgccgcgtggaggagctgcacagcaacaccgagctgggcatcgtggagtaccagcacgccttcaagacccccatcgccttcgccagatctcgagatatcagccatggcttcccgccggcggtggcggcgcaggatgatggcacgctgcccatgtcttgtgcccaggagagcgggatggaccgtcaccctgcagcctgtgcttctgctaggatcaatgtgaccggtgagggcagaggaagtcttctaacatgcggtgacgtggaggagaatcccggcccttccggtatggaagacgccaaaaacataaagaaaggcccggcgccattctatccgctagaggatggaaccgctggagagcaactgcataaggctatgaagagatacgccctggttcctggaacaattgcttttacagatgcacatatcgaggtgaacatcacgtacgcggaatacttcgaaatgtccgttcggttggcagaagctatgaaacgatatgggctgaatacaaatcacagaatcgtcgtatgcagtgaaaactctcttcaattctttatgccggtgttgggcgcgttatttatcggagttgcagttgcgcccgcgaacgacatttataatgaacgtgaattgctcaacagtatgaacatttcgcagcctaccgtagtgtttgtttccaaaaaggggttgcaaaaaattttgaacgtgcaaaaaaaattaccaataatccagaaaattattatcatggattctaaaacggattaccagggatttcagtcgatgtacacgttcgtcacatctcatctacctcccggttttaatgaatacgattttgtaccagagtcctttgatcgtgacaaaacaattgcactgataatgaactcctctggatctactgggttacctaagggtgtggcccttccgcatagaactgcctgcgtcagattctcgcatgccagagatcctatttttggcaatcaaatcattccggatactgcgattttaagtgttgttccattccatcacggttttggaatgtttactacactcggatatttgatatgtggatttcgagtcgtcttaatgtatagatttgaagaagagctgtttttacgatcccttcaggattacaaaattcaaagtgcgttgctagtaccaaccctattttcattcttcgccaaaagcactctgattgacaaatacgatttatctaatttacacgaaattgcttctgggggcgcacctctttcgaaagaagtcggggaagcggttgcaaaacgcttccatcttccagggatacgacaaggatatgggctcactgagactacatcagctattctgattacacccgagggggatgataaaccgggcgcggtcggtaaagttgttccattttttgaagcgaaggttgtggatctggataccgggaaaacgctgggcgttaatcagagaggcgaattatgtgtcagaggacctatgattatgtccggttatgtaaacaatccggaagcgaccaacgccttgattgacaaggatggatggctacattctggagacatagcttactgggacgaagacgaacacttcttcatagttgaccgcttgaagtctttaattaaatacaaaggataccaggtggcccccgctgaattggagtcgatattgttacaacaccccaacatcttcgacgcgggcgtggcaggtcttcccgacgatgacgccggtgaacttcccgccgccgttgttgttttggagcacggaaagacgatgacggaaaaagagatcgtggattacgtcgccagtcaagtaacaaccgcgaaaaagttgcgcggaggagttgtgtttgtggacgaagtaccgaaaggtcttaccggaaaactcgacgcaagaaaaatcagagagatcctcataaaggccaagaagggcggaaagtccaaattgtaatcgactcgacaatcaacctctggattcaagtaaagcggccgcgtcgactagagctcgctgatcagcctcgactgtgccttctagttgccagccatctgttgtttgcccctcccccgtgccttccttgaccctggaaggtgccactcccactgtcctttcctaataaaatgaggaaattgcatcgcattgtctgagtaggtgtcattctattctggggggtggggtggggcaggacagcaagggggaggattgggaagacaatagcaggcatgctggggagagatcactcgagctagccgcggcgcgccatggcggccgcttaattaatgcatcgcgatcgatgatatcagatctttacgtcacgactccacccctccaggaacccctagtgatggagttggccactccctctctgcgcgctcgctcgctcactgaggccgcccgggcaaagcccgggcgtcgggcgacctttggtcgcccggcctcagtgagcgagcgagcgcgcagagagggagtggccaa

>ITR-mCMV-GFP-T2A-Luc-PolyA-ITR to monitor inflammation pathway signaling (NF-kB)

ttggccactccctctctgcgcgctcgctcgctcactgaggccgggcgaccaaaggtcgcccgacgcccgggctttgcccgggcggcctcagtgagcgagcgagcgcgcagagagggagtggccaactccatcactaggggttccttgtagttaatgattaacccgccatgctacttatctacgtaacagatctgatatcacgcgtgtacactagtaccggtcctaggctcgagctccaagtgcaggggaaagaatagtagacataatagcaacagacatacaaactaaagaattacaaaaacaaattacaaaattcaaaattttatcgatgaattcctctagaaactagttaggcgtgtacggtgggaggcctatataagcagagctcgtttagtgaaccgtcagatcgcctggagacgccatccacgctgttttgacctccatagaagacaccgggaccgatccagcctctcgacattcgttggatccgccaccatgcccgccatgaagatcgagtgccgcatcaccggcaccctgaacggcgtggagttcgagctggtgggcggcggagagggcacccccgagcagggccgcatgaccaacaagatgaagagcaccaaaggcgccctgaccttcagcccctacctgctgagccacgtgatgggctacggcttctaccacttcggcacctaccccagcggctacgagaaccccttcctgcacgccatcaacaacggcggctacaccaacacccgcatcgagaagtacgaggacggcggcgtgctgcacgtgagcttcagctaccgctacgaggccggccgcgtgatcggcgacttcaaggtggtgggcaccggcttccccgaggacagcgtgatcttcaccgacaagatcatccgcagcaacgccaccgtggagcacctgcaccccatgggcgataacgtgctggtgggcagcttcgcccgcaccttcagcctgcgcgacggcggctactacagcttcgtggtggacagccacatgcacttcaagagcgccatccaccccagcatcctgcagaacgggggccccatgttcgccttccgccgcgtggaggagctgcacagcaacaccgagctgggcatcgtggagtaccagcacgccttcaagacccccatcgccttcgccagatctcgagatatcagccatggcttcccgccggcggtggcggcgcaggatgatggcacgctgcccatgtcttgtgcccaggagagcgggatggaccgtcaccctgcagcctgtgcttctgctaggatcaatgtgaccggtgagggcagaggaagtcttctaacatgcggtgacgtggaggagaatcccggcccttccggtatggaagacgccaaaaacataaagaaaggcccggcgccattctatccgctagaggatggaaccgctggagagcaactgcataaggctatgaagagatacgccctggttcctggaacaattgcttttacagatgcacatatcgaggtgaacatcacgtacgcggaatacttcgaaatgtccgttcggttggcagaagctatgaaacgatatgggctgaatacaaatcacagaatcgtcgtatgcagtgaaaactctcttcaattctttatgccggtgttgggcgcgttatttatcggagttgcagttgcgcccgcgaacgacatttataatgaacgtgaattgctcaacagtatgaacatttcgcagcctaccgtagtgtttgtttccaaaaaggggttgcaaaaaattttgaacgtgcaaaaaaaattaccaataatccagaaaattattatcatggattctaaaacggattaccagggatttcagtcgatgtacacgttcgtcacatctcatctacctcccggttttaatgaatacgattttgtaccagagtcctttgatcgtgacaaaacaattgcactgataatgaactcctctggatctactgggttacctaagggtgtggcccttccgcatagaactgcctgcgtcagattctcgcatgccagagatcctatttttggcaatcaaatcattccggatactgcgattttaagtgttgttccattccatcacggttttggaatgtttactacactcggatatttgatatgtggatttcgagtcgtcttaatgtatagatttgaagaagagctgtttttacgatcccttcaggattacaaaattcaaagtgcgttgctagtaccaaccctattttcattcttcgccaaaagcactctgattgacaaatacgatttatctaatttacacgaaattgcttctgggggcgcacctctttcgaaagaagtcggggaagcggttgcaaaacgcttccatcttccagggatacgacaaggatatgggctcactgagactacatcagctattctgattacacccgagggggatgataaaccgggcgcggtcggtaaagttgttccattttttgaagcgaaggttgtggatctggataccgggaaaacgctgggcgttaatcagagaggcgaattatgtgtcagaggacctatgattatgtccggttatgtaaacaatccggaagcgaccaacgccttgattgacaaggatggatggctacattctggagacatagcttactgggacgaagacgaacacttcttcatagttgaccgcttgaagtctttaattaaatacaaaggataccaggtggcccccgctgaattggagtcgatattgttacaacaccccaacatcttcgacgcgggcgtggcaggtcttcccgacgatgacgccggtgaacttcccgccgccgttgttgttttggagcacggaaagacgatgacggaaaaagagatcgtggattacgtcgccagtcaagtaacaaccgcgaaaaagttgcgcggaggagttgtgtttgtggacgaagtaccgaaaggtcttaccggaaaactcgacgcaagaaaaatcagagagatcctcataaaggccaagaagggcggaaagtccaaattgtaatcgactcgacaatcaacctctggattcaagtaaagcggccgcgtcgactagagctcgctgatcagcctcgactgtgccttctagttgccagccatctgttgtttgcccctcccccgtgccttccttgaccctggaaggtgccactcccactgtcctttcctaataaaatgaggaaattgcatcgcattgtctgagtaggtgtcattctattctggggggtggggtggggcaggacagcaagggggaggattgggaagacaatagcaggcatgctggggagagatcactcgagctagccgcggcgcgccatggcggccgcttaattaatgcatcgcgatcgatgatatcagatctttacgtcacgactccacccctccaggaacccctagtgatggagttggccactccctctctgcgcgctcgctcgctcactgaggccgcccgggcaaagcccgggcgtcgggcgacctttggtcgcccggcctcagtgagcgagcgagcgcgcagagagggagtggccaa

>ITR-CMV-GFP-T2A-Luc-PolyA-ITR to monitor inflammation pathway signaling (NF-kB)

ttggccactccctctctgcgcgctcgctcgctcactgaggccgggcgaccaaaggtcgcccgacgcccgggctttgcccgggcggcctcagtgagcgagcgagcgcgcagagagggagtggccaactccatcactaggggttccttgtagttaatgattaacccgccatgctacttatctacgtaacagatctgatatcacgcgtgtacactagtaccggtcctaggctcgagctccaagtgcaggggaaagaatagtagacataatagcaacagacatacaaactaaagaattacaaaaacaaattacaaaattcaaaattttatcgatgaattcGCGTTGACATTGATTATTGACTAGTTATTAATAGTAATCAATTACGGGGTCATTAGTTCATAGCCCATATATGGAGTTCCGCGTTACATAACTTACGGTAAATGGCCCGCCTGGCTGACCGCCCAACGACCCCCGCCCATTGACGTCAATAATGACGTATGTTCCCATAGTAACGCCAATAGGGACTTTCCATTGACGTCAATGGGTGGAGTATTTACGGTAAACTGCCCACTTGGCAGTACATCAAGTGTATCATATGCCAAGTACGCCCCCTATTGACGTCAATGACGGTAAATGGCCCGCCTGGCATTATGCCCAGTACATGACCTTATGGGACTTTCCTACTTGGCAGTACATCTACGTATTAGTCATCGCTATTACCATGGTGATGCGGTTTTGGCAGTACATCAATGGGCGTGGATAGCGGTTTGACTCACGGGGATTTCCAAGTCTCCACCCCATTGACGTCAATGGGAGTTTGTTTTGGCACCAAAATCAACGGGACTTTCCAAAATGTCGTAACAACTCCGCCCCATTGACGCAAATGGGCGGTAGGCGTGTACGGTGGGAGGTCTATATAAGCAGAGCTCTCTGGCTAACTACTAGTtaggcgtgtacggtgggaggcctatataagcagagctcgtttagtgaaccgtcagatcgcctggagacgccatccacgctgttttgacctccatagaagacaccgggaccgatccagcctctcgacattcgttggatccgccaccatgcccgccatgaagatcgagtgccgcatcaccggcaccctgaacggcgtggagttcgagctggtgggcggcggagagggcacccccgagcagggccgcatgaccaacaagatgaagagcaccaaaggcgccctgaccttcagcccctacctgctgagccacgtgatgggctacggcttctaccacttcggcacctaccccagcggctacgagaaccccttcctgcacgccatcaacaacggcggctacaccaacacccgcatcgagaagtacgaggacggcggcgtgctgcacgtgagcttcagctaccgctacgaggccggccgcgtgatcggcgacttcaaggtggtgggcaccggcttccccgaggacagcgtgatcttcaccgacaagatcatccgcagcaacgccaccgtggagcacctgcaccccatgggcgataacgtgctggtgggcagcttcgcccgcaccttcagcctgcgcgacggcggctactacagcttcgtggtggacagccacatgcacttcaagagcgccatccaccccagcatcctgcagaacgggggccccatgttcgccttccgccgcgtggaggagctgcacagcaacaccgagctgggcatcgtggagtaccagcacgccttcaagacccccatcgccttcgccagatctcgagatatcagccatggcttcccgccggcggtggcggcgcaggatgatggcacgctgcccatgtcttgtgcccaggagagcgggatggaccgtcaccctgcagcctgtgcttctgctaggatcaatgtgaccggtgagggcagaggaagtcttctaacatgcggtgacgtggaggagaatcccggcccttccggtatggaagacgccaaaaacataaagaaaggcccggcgccattctatccgctagaggatggaaccgctggagagcaactgcataaggctatgaagagatacgccctggttcctggaacaattgcttttacagatgcacatatcgaggtgaacatcacgtacgcggaatacttcgaaatgtccgttcggttggcagaagctatgaaacgatatgggctgaatacaaatcacagaatcgtcgtatgcagtgaaaactctcttcaattctttatgccggtgttgggcgcgttatttatcggagttgcagttgcgcccgcgaacgacatttataatgaacgtgaattgctcaacagtatgaacatttcgcagcctaccgtagtgtttgtttccaaaaaggggttgcaaaaaattttgaacgtgcaaaaaaaattaccaataatccagaaaattattatcatggattctaaaacggattaccagggatttcagtcgatgtacacgttcgtcacatctcatctacctcccggttttaatgaatacgattttgtaccagagtcctttgatcgtgacaaaacaattgcactgataatgaactcctctggatctactgggttacctaagggtgtggcccttccgcatagaactgcctgcgtcagattctcgcatgccagagatcctatttttggcaatcaaatcattccggatactgcgattttaagtgttgttccattccatcacggttttggaatgtttactacactcggatatttgatatgtggatttcgagtcgtcttaatgtatagatttgaagaagagctgtttttacgatcccttcaggattacaaaattcaaagtgcgttgctagtaccaaccctattttcattcttcgccaaaagcactctgattgacaaatacgatttatctaatttacacgaaattgcttctgggggcgcacctctttcgaaagaagtcggggaagcggttgcaaaacgcttccatcttccagggatacgacaaggatatgggctcactgagactacatcagctattctgattacacccgagggggatgataaaccgggcgcggtcggtaaagttgttccattttttgaagcgaaggttgtggatctggataccgggaaaacgctgggcgttaatcagagaggcgaattatgtgtcagaggacctatgattatgtccggttatgtaaacaatccggaagcgaccaacgccttgattgacaaggatggatggctacattctggagacatagcttactgggacgaagacgaacacttcttcatagttgaccgcttgaagtctttaattaaatacaaaggataccaggtggcccccgctgaattggagtcgatattgttacaacaccccaacatcttcgacgcgggcgtggcaggtcttcccgacgatgacgccggtgaacttcccgccgccgttgttgttttggagcacggaaagacgatgacggaaaaagagatcgtggattacgtcgccagtcaagtaacaaccgcgaaaaagttgcgcggaggagttgtgtttgtggacgaagtaccgaaaggtcttaccggaaaactcgacgcaagaaaaatcagagagatcctcataaaggccaagaagggcggaaagtccaaattgtaatcgactcgacaatcaacctctggattcaagtaaagcggccgcgtcgactagagctcgctgatcagcctcgactgtgccttctagttgccagccatctgttgtttgcccctcccccgtgccttccttgaccctggaaggtgccactcccactgtcctttcctaataaaatgaggaaattgcatcgcattgtctgagtaggtgtcattctattctggggggtggggtggggcaggacagcaagggggaggattgggaagacaatagcaggcatgctggggagagatcactcgagctagccgcggcgcgccatggcggccgcttaattaatgcatcgcgatcgatgatatcagatctttacgtcacgactccacccctccaggaacccctagtgatggagttggccactccctctctgcgcgctcgctcgctcactgaggccgcccgggcaaagcccgggcgtcgggcgacctttggtcgcccggcctcagtgagcgagcgagcgcgcagagagggagtggccaa

>ITR-NFϰB-TRE-mCMV-GFP-T2A-Luc-PolyA-ITR to monitor inflammation pathway signaling (NF-ϰB)

ttggccactccctctctgcgcgctcgctcgctcactgaggccgggcgaccaaaggtcgcccgacgcccgggctttgcccgggcggcctcagtgagcgagcgagcgcgcagagagggagtggccaactccatcactaggggttccttgtagttaatgattaacccgccatgctacttatctacgtaacagatctgatatcacgcgtgtacactagtaccggtcctaggctcgagctccaagtgcaggggaaagaatagtagacataatagcaacagacatacaaactaaagaattacaaaaacaaattacaaaattcaaaattttatcgatgaattcGGGACTTTCCATTGTAGCGTAGGGACTTTCCATTGTAGCGTAGGGACTTTCCATTGTAGCGTAGGGACTTTCCATTGTAGCGTAactagttaggcgtgtacggtgggaggcctatataagcagagctcgtttagtgaaccgtcagatcgcctggagacgccatccacgctgttttgacctccatagaagacaccgggaccgatccagcctctcgacattcgttggatccgccaccatgcccgccatgaagatcgagtgccgcatcaccggcaccctgaacggcgtggagttcgagctggtgggcggcggagagggcacccccgagcagggccgcatgaccaacaagatgaagagcaccaaaggcgccctgaccttcagcccctacctgctgagccacgtgatgggctacggcttctaccacttcggcacctaccccagcggctacgagaaccccttcctgcacgccatcaacaacggcggctacaccaacacccgcatcgagaagtacgaggacggcggcgtgctgcacgtgagcttcagctaccgctacgaggccggccgcgtgatcggcgacttcaaggtggtgggcaccggcttccccgaggacagcgtgatcttcaccgacaagatcatccgcagcaacgccaccgtggagcacctgcaccccatgggcgataacgtgctggtgggcagcttcgcccgcaccttcagcctgcgcgacggcggctactacagcttcgtggtggacagccacatgcacttcaagagcgccatccaccccagcatcctgcagaacgggggccccatgttcgccttccgccgcgtggaggagctgcacagcaacaccgagctgggcatcgtggagtaccagcacgccttcaagacccccatcgccttcgccagatctcgagatatcagccatggcttcccgccggcggtggcggcgcaggatgatggcacgctgcccatgtcttgtgcccaggagagcgggatggaccgtcaccctgcagcctgtgcttctgctaggatcaatgtgaccggtgagggcagaggaagtcttctaacatgcggtgacgtggaggagaatcccggcccttccggtatggaagacgccaaaaacataaagaaaggcccggcgccattctatccgctagaggatggaaccgctggagagcaactgcataaggctatgaagagatacgccctggttcctggaacaattgcttttacagatgcacatatcgaggtgaacatcacgtacgcggaatacttcgaaatgtccgttcggttggcagaagctatgaaacgatatgggctgaatacaaatcacagaatcgtcgtatgcagtgaaaactctcttcaattctttatgccggtgttgggcgcgttatttatcggagttgcagttgcgcccgcgaacgacatttataatgaacgtgaattgctcaacagtatgaacatttcgcagcctaccgtagtgtttgtttccaaaaaggggttgcaaaaaattttgaacgtgcaaaaaaaattaccaataatccagaaaattattatcatggattctaaaacggattaccagggatttcagtcgatgtacacgttcgtcacatctcatctacctcccggttttaatgaatacgattttgtaccagagtcctttgatcgtgacaaaacaattgcactgataatgaactcctctggatctactgggttacctaagggtgtggcccttccgcatagaactgcctgcgtcagattctcgcatgccagagatcctatttttggcaatcaaatcattccggatactgcgattttaagtgttgttccattccatcacggttttggaatgtttactacactcggatatttgatatgtggatttcgagtcgtcttaatgtatagatttgaagaagagctgtttttacgatcccttcaggattacaaaattcaaagtgcgttgctagtaccaaccctattttcattcttcgccaaaagcactctgattgacaaatacgatttatctaatttacacgaaattgcttctgggggcgcacctctttcgaaagaagtcggggaagcggttgcaaaacgcttccatcttccagggatacgacaaggatatgggctcactgagactacatcagctattctgattacacccgagggggatgataaaccgggcgcggtcggtaaagttgttccattttttgaagcgaaggttgtggatctggataccgggaaaacgctgggcgttaatcagagaggcgaattatgtgtcagaggacctatgattatgtccggttatgtaaacaatccggaagcgaccaacgccttgattgacaaggatggatggctacattctggagacatagcttactgggacgaagacgaacacttcttcatagttgaccgcttgaagtctttaattaaatacaaaggataccaggtggcccccgctgaattggagtcgatattgttacaacaccccaacatcttcgacgcgggcgtggcaggtcttcccgacgatgacgccggtgaacttcccgccgccgttgttgttttggagcacggaaagacgatgacggaaaaagagatcgtggattacgtcgccagtcaagtaacaaccgcgaaaaagttgcgcggaggagttgtgtttgtggacgaagtaccgaaaggtcttaccggaaaactcgacgcaagaaaaatcagagagatcctcataaaggccaagaagggcggaaagtccaaattgtaatcgactcgacaatcaacctctggattcaagtaaagcggccgcgtcgactagagctcgctgatcagcctcgactgtgccttctagttgccagccatctgttgtttgcccctcccccgtgccttccttgaccctggaaggtgccactcccactgtcctttcctaataaaatgaggaaattgcatcgcattgtctgagtaggtgtcattctattctggggggtggggtggggcaggacagcaagggggaggattgggaagacaatagcaggcatgctggggagagatcactcgagctagccgcggcgcgccatggcggccgcttaattaatgcatcgcgatcgatgatatcagatctttacgtcacgactccacccctccaggaacccctagtgatggagttggccactccctctctgcgcgctcgctcgctcactgaggccgcccgggcaaagcccgggcgtcgggcgacctttggtcgcccggcctcagtgagcgagcgagcgcgcagagagggagtggccaa

>ITR-AP1-TRE-mCMV-GFP-T2A-Luc-PolyA-ITR to monitor MAPK growth pathway signaling

ttggccactccctctctgcgcgctcgctcgctcactgaggccgggcgaccaaaggtcgcccgacgcccgggctttgcccgggcggcctcagtgagcgagcgagcgcgcagagagggagtggccaactccatcactaggggttccttgtagttaatgattaacccgccatgctacttatctacgtaacagatctgatatcacgcgtgtacactagtaccggtcctaggctcgagctccaagtgcaggggaaagaatagtagacataatagcaacagacatacaaactaaagaattacaaaaacaaattacaaaattcaaaattttatcgatgaattcGGTGACTCAGTGACTGAGTCAGATAGGTGACTCAGTGACTGAGTCAGATAGGTGACTCAGTGACTGAGTCAGATAGGTGACTCAGTGACTGAGTCAGATAactagttaggcgtgtacggtgggaggcctatataagcagagctcgtttagtgaaccgtcagatcgcctggagacgccatccacgctgttttgacctccatagaagacaccgggaccgatccagcctctcgacattcgttggatccgccaccatgcccgccatgaagatcgagtgccgcatcaccggcaccctgaacggcgtggagttcgagctggtgggcggcggagagggcacccccgagcagggccgcatgaccaacaagatgaagagcaccaaaggcgccctgaccttcagcccctacctgctgagccacgtgatgggctacggcttctaccacttcggcacctaccccagcggctacgagaaccccttcctgcacgccatcaacaacggcggctacaccaacacccgcatcgagaagtacgaggacggcggcgtgctgcacgtgagcttcagctaccgctacgaggccggccgcgtgatcggcgacttcaaggtggtgggcaccggcttccccgaggacagcgtgatcttcaccgacaagatcatccgcagcaacgccaccgtggagcacctgcaccccatgggcgataacgtgctggtgggcagcttcgcccgcaccttcagcctgcgcgacggcggctactacagcttcgtggtggacagccacatgcacttcaagagcgccatccaccccagcatcctgcagaacgggggccccatgttcgccttccgccgcgtggaggagctgcacagcaacaccgagctgggcatcgtggagtaccagcacgccttcaagacccccatcgccttcgccagatctcgagatatcagccatggcttcccgccggcggtggcggcgcaggatgatggcacgctgcccatgtcttgtgcccaggagagcgggatggaccgtcaccctgcagcctgtgcttctgctaggatcaatgtgaccggtgagggcagaggaagtcttctaacatgcggtgacgtggaggagaatcccggcccttccggtatggaagacgccaaaaacataaagaaaggcccggcgccattctatccgctagaggatggaaccgctggagagcaactgcataaggctatgaagagatacgccctggttcctggaacaattgcttttacagatgcacatatcgaggtgaacatcacgtacgcggaatacttcgaaatgtccgttcggttggcagaagctatgaaacgatatgggctgaatacaaatcacagaatcgtcgtatgcagtgaaaactctcttcaattctttatgccggtgttgggcgcgttatttatcggagttgcagttgcgcccgcgaacgacatttataatgaacgtgaattgctcaacagtatgaacatttcgcagcctaccgtagtgtttgtttccaaaaaggggttgcaaaaaattttgaacgtgcaaaaaaaattaccaataatccagaaaattattatcatggattctaaaacggattaccagggatttcagtcgatgtacacgttcgtcacatctcatctacctcccggttttaatgaatacgattttgtaccagagtcctttgatcgtgacaaaacaattgcactgataatgaactcctctggatctactgggttacctaagggtgtggcccttccgcatagaactgcctgcgtcagattctcgcatgccagagatcctatttttggcaatcaaatcattccggatactgcgattttaagtgttgttccattccatcacggttttggaatgtttactacactcggatatttgatatgtggatttcgagtcgtcttaatgtatagatttgaagaagagctgtttttacgatcccttcaggattacaaaattcaaagtgcgttgctagtaccaaccctattttcattcttcgccaaaagcactctgattgacaaatacgatttatctaatttacacgaaattgcttctgggggcgcacctctttcgaaagaagtcggggaagcggttgcaaaacgcttccatcttccagggatacgacaaggatatgggctcactgagactacatcagctattctgattacacccgagggggatgataaaccgggcgcggtcggtaaagttgttccattttttgaagcgaaggttgtggatctggataccgggaaaacgctgggcgttaatcagagaggcgaattatgtgtcagaggacctatgattatgtccggttatgtaaacaatccggaagcgaccaacgccttgattgacaaggatggatggctacattctggagacatagcttactgggacgaagacgaacacttcttcatagttgaccgcttgaagtctttaattaaatacaaaggataccaggtggcccccgctgaattggagtcgatattgttacaacaccccaacatcttcgacgcgggcgtggcaggtcttcccgacgatgacgccggtgaacttcccgccgccgttgttgttttggagcacggaaagacgatgacggaaaaagagatcgtggattacgtcgccagtcaagtaacaaccgcgaaaaagttgcgcggaggagttgtgtttgtggacgaagtaccgaaaggtcttaccggaaaactcgacgcaagaaaaatcagagagatcctcataaaggccaagaagggcggaaagtccaaattgtaatcgactcgacaatcaacctctggattcaagtaaagcggccgcgtcgactagagctcgctgatcagcctcgactgtgccttctagttgccagccatctgttgtttgcccctcccccgtgccttccttgaccctggaaggtgccactcccactgtcctttcctaataaaatgaggaaattgcatcgcattgtctgagtaggtgtcattctattctggggggtggggtggggcaggacagcaagggggaggattgggaagacaatagcaggcatgctggggagagatcactcgagctagccgcggcgcgccatggcggccgcttaattaatgcatcgcgatcgatgatatcagatctttacgtcacgactccacccctccaggaacccctagtgatggagttggccactccctctctgcgcgctcgctcgctcactgaggccgcccgggcaaagcccgggcgtcgggcgacctttggtcgcccggcctcagtgagcgagcgagcgcgcagagagggagtggccaa

Note: TRE: transcription factor response element
